# A case of Lemierre syndrome

**DOI:** 10.4103/0256-4947.51822

**Published:** 2009

**Authors:** Ameen Alherabi

**Affiliations:** From the Department of Otolaryngology/Head & Neck Surgery, Umm Al-Qura University and Al-Noor Specialist Hospital, Makkah, Saudi Arabia

Lemierre syndrome, also known as postanginal septicemia or necrobacillosis was first reported in 1890 by Courmont and Cade, although Lemierre, a French physician and professor of microbiology best described the syndrome in 1936 in a review of 20 cases that was published in the Lancet.^1^ His cases were based on the following criteria: (1) acute oropharyngeal infection or abscess, (2) septicemia, (3) throm-bophlebitis of the internal jugular vein and (4) secondary metastatic abscesses, most commonly to the lungs and joints.^1^,^2^ It is seen mostly in previously well adolescents and young adults with a mean age of 19 years, and there is a slightly higher prevalence in males. The current mortality rate is estimated to be between 5% and 10%, with significant morbidity.^3^–^5^ Before the advent of antibiotics, this syndrome was commonly encountered and often fatal.^1^ Presumably, owing to the widespread use of antibiotics in the early treatment of throat infections, there has been a dramatic decrease in the incidence of Lemierre syndrome; fewer than 100 cases have been reported since 1974.^6^,^7^

## CASE

**Figure 1:**
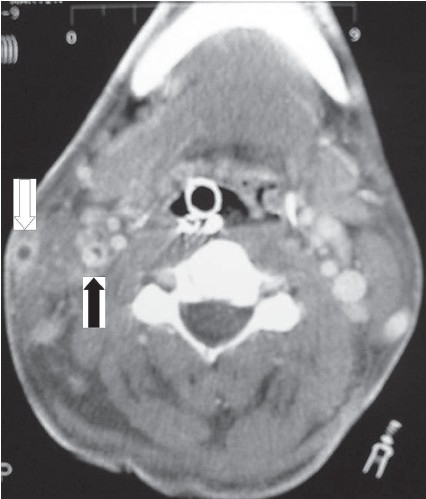
CT of the neck showing partial thrombosis of the right internal jugular vein (black arrow) and complete thrombosis of the right external jugular vein (white arrow).

A 27-year-old otherwise healthy male presented to the emergency department with a 10-day history of sore throat, fever and lymphadenopathy. His condition had deteriorated to significant odynophagia, hemoptysis, chest pain and shortness of breath. On examination his temperature was 38°C, oxygen saturation was 93% on 2 liters of oxygen. He was jaundiced and had tender cervical lymphadenopathy and an erythrematous throat. His chest exam showed bilateral bibasilar crackles, and he had hepatosplenomegaly. His initial investigation showed WBC 18.9×10^9^ Lwith neutrophil count of 15.69×10^9^ L and a platelet count of 23/mm^3^. Platelets 23. A chest x-ray showed evidence of pneumonia and pleural effusion. He was treated as presumed pneumonia with IV antibiotics, but his condition deteriorated to respiratory failure and sepsis requiring mechanical ventilation. A CT scan showed bilateral pulmonary empyema and thrombosed jugular veins (Figure 1). Blood culture grew *Fusobacterium necrophorum.* The diagnosis of Lemierre syndrome was made. The patient was treated with IV penicillin and required a bilateral thoracotomy and surgical evacuation of the chest empyema. His condition improved and he was extubated and sent home to continue a 6-week course of oral penicillin.

## DISCUSSION

*Fusobacterium* species are normal inhabitants of the oral cavity, the female genital tract, and the gastrointestinal tract. Of this species, *F nucleatum* and *F necrophorum* are the most commonly isolated. They are slow growing, strictly anaerobic, gram-negative pleomorphic bacilli. *F necrophorum,* the most common pathogen, has an ability to invade as a primary pathogen. This feature is related to the bacteria's ability to produce lipopolysac-charide endotoxin, leukocidin, and haemolysin. Other organisms isolated from patients with this syndrome include *Bacteroides, Streptococcus, Peptostreptococcus* and *Eikenella corrodens,* with more than one pathogen being reported in a few cases.^8^–^18^

The principal source of infection is located in the palatine tonsils and peritonsillar tissue, but other sources (parotitis, otitis media, sinusitis, odontogenic infection and mastoid) have been reported.^16^,^19^–^21^ Subsequently, the parapharyngeal space may become infected. Secondary thrombophlebitis of the tonsillar veins may occur owing to a pharyngeal infection, which can propagate thrombus formation to the internal jugular vein (IJV).^4^,^7^,^22^ Alternatively, direct extension is another route of access to the IJV. More specifically, oropharyngeal infection and abscess formation may travel directly to the parapharyngeal space, hence, direct extension from the submandibular, peritonsillar, retropharyngeal, parotid and masticator spaces may lead to IJV thrombosis.^23^–^24^

The time of onset from the initial oropharyngitis to the development of septicemia is usually less than 7 days.^1^,^25^–^26^ The primary source of infection is usually the palatine tonsils, with most patients presenting with exudative tonsillitis or peritonsillar abscesses.^7^–^8^,^14^ The development of thrombophlebitis of the IJV is associated with neck pain and swelling. This may occasionally be associated with trismus, otalgia and dysphagia.^27^ A relatively recent case similar to ours showed involvement of branches of the external jugular vein in addition to the IJV.^23^ The most common site of metastatic infection is the lungs. This may be associated with bilateral pulmonary infiltrates, pleural effusion, empyema and lung abscess or multiple cavitating lung lesions.^16^,^20^,^24^,^28^,^29^ Other complications described include pneumatoceles, pneumothorax and adult respiratory distress syndrome. 30–32 Other manifestations of metastatic infection include septic arthritis, osteomyelitis, pericarditis, hepatic abscesses and meningitis. Moreover, the spleen, skin, kidneys, brain and soft tissues may be involved.^16^,^19^,^21^,^33^ Splenomegaly, hepatomegaly and jaundice may be present, along with pain associated with the involved metastatic sites. Milde-to-moderate hyperbilirubinemia with elevated liver enzymes is commonly found. The blood count usually shows leukocytosis and a few cases exhibiting thrombocytopenia have been noted. Renal insufficiency with hematuria, pyuria, proteinuria, and/or a rise in blood urea may result from kidney involvement.^3^,^15^,^18^,^34^ Coagulopathy and disseminated intravascular coagulation also has been reported.^31^ Fatality is rare in the post-antibiotic era.^12^,^13^

A high degree of suspicion is needed and usually the syndrome is not diagnosed until microbiology results are available.^10^,^17^ With suspected clinical picture imaging modalities usually confirm the diagnosis. Contrast-enhanced CT provides exceptional accuracy in the diagnosis of Lemierre syndrome because of its ability to show distended veins with enhancement of the walls, intramural filling defects and swelling of adjacent soft tissues, which allows the delineation of additional pathology (e.g. abscess extension) and sensitive visualization of the intrathoracic veins.^35^ Other alternatives include Doppler ultrasound, MRA, gallium scan and radionuclide venography with Tc 99m-labeled RBCs. Conventional retrograde venography is currently reserved for assessing the exact extent of IJV thrombus when surgical ligation is considered. Chest x-ray and CT are used to diagnose pulmonary findings. Abdominal ultrasound is employed when hepatic or splenic abscesses are suspected.^35^–^39^ The organism is usually isolated from blood cultures. Other microbiological specimens include synovial fluid, skin pustules and pus from liver abscesses, empyema and a bronchoscopic aspirate.^10^,^17^

Intravenous antibiotics are the mainstay treatment. *F necrophorum* is usually sensitive to penicillin, clindamycin and metronidazole.^10^,^14^–^15^,^17^ Clinical improvement after the onset of antibiotic therapy is sometimes slow, yet numerous cases have demonstrated a full recovery being achieved with the same antibiotic treatment after a brief period of apparently unfavorable evolution.^3^–^4^,^26^

The majority of patients treated with appropriate antibiotics have a favorable prognosis, but delayed treatment is associated with poorer outcomes. Prolonged therapy is recommended because of the endovascular nature of the infection, with most reports indicating a good clinical response within 2 to 9 weeks of therapy with a mean of 6 weeks.^3^,^4^,^26^

Surgical excision and ligation of the IJV is hardly necessary nowadays and is only reserved for cases with continued sepsis and septic embolization resulting in severe respiratory compromise or other repetitive embolic manifestations.^4^,^7^–^9^,^22^–^23^

The role of anticoagulation in Lemierre syndrome remains controversial. Some studies reported the use of heparin being associated with expeditious resolution of the thrombophlebitis, which may shorten the course of the disease and may reduce the need for any surgical intervention. Therefore, anticoagulant therapy may be initiated for possible early clot dissolution, but should be discontinued in patients who require surgery.^8^,^40^ The clear indication for use of anticoagulation in Lemierre syndrome is sigmoid sinus thrombosis with possible retrograde propagation to the cavernous sinus and inferior vena cava thrombosis.^8^–^9^,^16^,^21^ When indicated, the recommended treatment regimen consists of one week of intravenous heparin followed by three months of oral warfarin.^8^,^21^,^40^ The use of steroids in the management of Lemierre syndrome has not been studied in a well organized scientific setting and there is no clear evidence to suggest a beneficial effect. There is some evidence to support a beneficial use of hyperbaric oxygen.^41^

A high degree of clinical suspicion is needed if symptoms from oropharyngeal infection or from any of the mentioned sources are accompanied or followed by a presentation suggestive of IJV thrombophlebitis, sepsis or septic emboli. Persistent fever may be the only physical evidence, particularly during the earlier phases of the disease.

Early recognition of the syndrome is crucial to allow the initiation of immediate appropriate therapy because microbiologic confirmation may take several days. Hence, we stress the importance of taking early blood cultures and carrying out a careful examination of the neck in suspicious patients presenting with a severe oropharyngitis. Also, contrast-enhanced CT should be performed as early as possible because physical examination of the neck and ultrasonography may be negative for thrombosis of the IJV in some cases.
